# Impact of HLA Selection Pressure on HIV Fitness at a Population Level in Mexico and Barbados

**DOI:** 10.1128/JVI.01162-14

**Published:** 2014-09

**Authors:** Claudia I. Juarez-Molina, Rebecca Payne, Maribel Soto-Nava, Santiago Avila-Rios, Humberto Valenzuela-Ponce, Emily Adland, Ellen Leitman, Jacqui Brener, Maximilian Muenchhoff, Songee Branch, Clive Landis, Gustavo Reyes-Teran, Philip Goulder

**Affiliations:** a Department of Paediatrics, University of Oxford, Oxford, United Kingdom; b Centre for Research in Infectious Diseases, National Institute of Respiratory Diseases, Mexico City, Mexico; c Ladymeade Reference Unit, Queen Elizabeth Hospital, Bridgetown, Barbados

## Abstract

Previous studies have demonstrated that effective cytotoxic T lymphocyte (CTL) responses drive the selection of escape mutations that reduce viral replication capacity (VRC). Escape mutations, including those with reduced VRC, can be transmitted and accumulate in a population. Here we compared two antiretroviral therapy (ART)-naive HIV clade B-infected cohorts, in Mexico and Barbados, in which the most protective HLA alleles (HLA-B*27/57/58:01/81:01) are differentially expressed, at 8% and 34%, respectively. Viral loads were significantly higher in Mexico than in Barbados (median, 40,774 versus 14,200; *P* < 0.0001), and absolute CD4^+^ T-cell counts were somewhat lower (median, 380/mm^3^ versus 403/mm^3^; *P* = 0.007). We tested the hypothesis that the disparate frequencies of these protective HLA alleles would be associated with a higher VRC at the population level in Mexico. Analysis of VRC in subjects in each cohort, matched for CD4^+^ T-cell count, revealed that the VRC was indeed higher in the Mexican cohort (mean, 1.13 versus 1.03; *P* = 0.0025). Although CD4 counts were matched, viral loads remained significantly higher in the Mexican subjects (*P* = 0.04). This VRC difference was reflected by a significantly higher frequency in the Barbados cohort of HLA-B*27/57/58:01/81:01-associated Gag escape mutations previously shown to incur a fitness cost on the virus (*P* = 0.004), a difference between the two cohorts that remained statistically significant even in subjects not expressing these protective alleles (*P* = 0.01). These data suggest that viral set points and disease progression rates at the population level may be significantly influenced by the prevalence of protective HLA alleles such as HLA-B*27/57/58:01/81:01 and that CD4 count-based guidelines to initiate antiretroviral therapy may need to be modified accordingly, to optimize the effectiveness of treatment-for-prevention strategies and reduce HIV transmission rates to the absolute minimum.

**IMPORTANCE** Immune control of HIV at an individual level is strongly influenced by the HLA class I genotype. HLA class I molecules mediating effective immune control, such as HLA-B*27 and HLA-B*57, are associated with the selection of escape mutants that reduce viral replicative capacity. The escape mutants selected in infected patients can be transmitted and affect the viral load and CD4 count in the recipient. These findings prompt the hypothesis that the frequency of protective alleles in a population may affect viral set points and rates of disease progression in that population. These studies in Mexico and Barbados, where the prevalence rates of protective HLA alleles are 8% and 34%, respectively, support this hypothesis. These data suggest that antiretroviral therapy (ART) treatment-for-prevention strategies will be less successful in populations such as those in Mexico, where viral loads are higher for a given CD4 count. Consideration may therefore usefully be given to ART initiation at higher absolute CD4 counts in such populations to optimize the impact of ART for prevention.

## INTRODUCTION

It is well established that HLA class I molecules exert strong selection pressure on HIV (1–3). Viral escape mutations selected in response to Gag-specific cytotoxic T lymphocyte (CTL) activity restricted by protective HLA molecules have fitness costs, as indicated either by *in vitro* fitness assays or by *in vivo* reversion following transmission to HLA-mismatched individuals (4–18). The population level frequency of escape variants, including those associated with reduced viral replicative capacity, appears to be accumulating, being strongly correlated with the prevalence of the relevant HLA allele in that population (19). Studies of linked transmission pairs have shown that CD4^+^ T-cell count and viral load in a newly infected individual are both strongly related to the replicative capacity of the transmitted virus (20–23). Together, these data prompt the hypotheses, first, that the impact of protective HLA molecules may bring about a reduction in population level viral fitness as the epidemic progresses, and, second, that marked differences in viral fitness may be evident in populations in which protective HLA alleles are expressed at differential levels.

Data supporting these hypotheses have come from Japan. Recent studies have indicated that viral replicative capacity is declining as the epidemic progresses in that country (24). Previously, it had been shown that HLA-B*51 was protective early in the Japanese epidemic but is now no longer protective (19). This loss of HLA-B*51-associated disease protection has been linked to an increase in the frequency of the escape mutation RT-135 (I135X) that abrogates HLA-B*51 binding to the epitope TAFTIPSI (RT-128 to -135). The HLA-B*51-TAFTIPSI response, in turn, has been associated with HLA-B*51-mediated immune control of HIV (25). These data therefore are consistent with the possibility that viral adaptation to protective HLA molecules may come at the cost of a decline in population level viral fitness through the accumulation of escape mutants that reduce viral replicative capacity.

**FIG 1:**
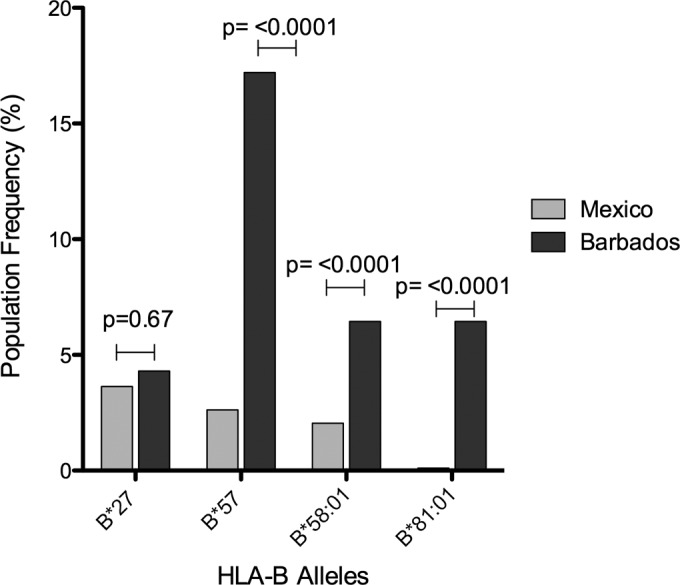
Population frequency of previously described protective class I HLA-B alleles for HIV clade B chronically infected individuals from Mexico (*n* = 976) and Barbados (*n* = 190). Prevalence of HLA-B*27, HLA-B*57, HLA-B*58:01, and HL:A-B*81:01 in Mexico is shown in grey and in Barbados in black.

To better understand the impact that protective HLA alleles may have on HIV adaptation at a population level, we compared two antiretroviral therapy (ART)-naïve HIV clade B-infected cohorts in which the frequency of protective alleles was substantially different. A low prevalence of the protective alleles HLA-B*27/57/58:01/81:01 (26) was observed in the studied Mexican cohort (8%, *n* = 926), in contrast to the Barbados cohort, exhibiting substantially higher prevalence of these alleles (34%, *n* = 190) (Fig. 1). We wished to test the hypotheses that first, the population level viral fitness might be lower in Barbados than in the Mexican cohort, and second, the frequency of escape mutations associated with fitness cost driven by HLA-B*27/57/58:01/81:01 is higher in Barbados.

## MATERIALS AND METHODS

### Study cohorts.

The study comprised two adult ART-naive HIV clade B-infected cohorts (27, 28). The Mexican cohort included 926 individuals recruited from the Centre for Research in Infectious Diseases, Mexico City, Mexico. These subjects were enrolled from 2011 to 2012. Viral load (VL) and CD4^+^ T-cell counts were available for 926 and 919 study subjects, respectively. The median VL of this cohort was 40,774 HIV RNA copies/ml plasma (interquartile range [IQR], 10,334 to 133,589 c/ml), and the median CD4^+^ T-cell count was 380 cells/mm^3^ (IQR, 151 to 592). The mean age of the cohort was 33 years, and the cohort comprised 20% females. The Barbados cohort included 190 individuals recruited from Bridgetown, Barbados. These subjects were enrolled from 2008 to 2010. VL and CD4^+^ T-cell counts were available for 176 and 171 study subjects, respectively. The median VL of this cohort was 14,200 HIV RNA copies/ml plasma (IQR, 2,775 to 59,275), and the median CD4^+^ T-cell count was 403 cells/mm^3^ (IQR, 325 to 569). The mean age of the cohort was 38 years, and the cohort comprised 44% females.

Ethical approval for the study was obtained from the Oxford Research Ethics Committee, the Institutional Bioethics and Science Committee in Mexico, and the Barbados Ministry of Health. Written informed consent was obtained from all individuals. In Barbados, viral load determinations were performed using the Roche Cobas Amplicor (Roche Diagnostics, Mannheim, Germany) from 2002 to October 2007 and Roche AmpliPrep and TaqMan 48 Analysers (Roche Diagnostics) from November 2007 to December 2011. In Mexico, viral loads were initially determined using the same Roche Ampli-Prep Cobas platform and then, beginning in 2009, using the Abbott m2000 platform. The Abbott m2000 platform underreports HIV-1 RNA levels compared to the Roche platforms, and therefore the difference in viral loads observed might if anything be an underestimate (see Discussion) (29, 30). CD4^+^ T-cell counts were determined by multiparametric flow cytometry (BD).

### HLA typing.

Four-digit HLA typing of the class I locus for the samples was performed from genomic DNA as previously described (31) by sequence-based typing. Samples from Barbados were typed at the American Society for Histocompatibility and Immunogenetics (ASHI)-accredited HLA typing laboratory, University of Oklahoma Health Sciences Center, Oklahoma City, OK, USA. Samples from Mexico were typed on-site, using the same method.

### Generation of Gag-protease NL4-3 recombinant viruses.

Gag-protease NL4-3 recombinant virus stocks were generated as previously described (32) by cotransfection of HIV-inducible, GXR-CEM cells with Gag-protease-deleted NL4-3 plasmid and clinically derived gag-protease amplicons from study subjects. The Gag-protease-deleted pNL4-3 (pNL4-3Δgag-protease) plasmid was kindly provided by Mark Brockman, Canada. In brief, viral RNA was isolated from plasma samples using the QIAamp viral RNA minikit from Qiagen (Valencia, CA). Reverse transcription-PCR (RT-PCR) was performed using the Superscript III One-Step RT-PCR kit (Invitrogen, Carlsbad, CA). A second round of PCR was performed with 100-mer forward and reverse primers complementary to NL4-3 on either side of Gag-protease, using a HighFidelity Platinum *Taq* polymerase (Invitrogen). For each sample, 10 μg of the pNL4-3Δgag-protease was linearized by BstE II (New England BioLabs, Ipswich, MA) digestion and cotransfected with 5 μg of purified PCR product of autologous Gag-protease from patients into 2 × 10^6^ GXR-CEM reporter cells in 300 μl of R10 medium. Electroporation was carried out at 250 V and 950 μF for 30 ms. The cells were left to rest for 5 min and transferred to a T25 flask containing 1 × 10^6^ GXR cells in 10 ml R10 medium. Cells were then incubated at 37°C. Green fluorescent protein (GFP) expression was measured every 2 days using a FACSCalibur flow cytometer (BD Biosciences, San Jose, CA) as per the manufacturer's instructions. Chimeric viruses were harvested when >15% of GXR-CEM cells were GFP positive (GFP^+^) and stored at −80°C. Viruses from patients with a CD4^+^ T-cell count between 300 and 500 cells/mm^3^ were selected for comparison between cohorts; 45 and 49 chimeric viruses from Mexico and Barbados, respectively, were studied.

### VRC assay.

Titration of virus stocks and replication assays were performed as previously described (5, 8), using a multiplicity of infection (MOI) of 0.003. All assays were carried out in Oxford using aliquots of the same NL4-3 viral stocks as the control to normalize measurements of viral replication capacity (VRC). In brief, titration was carried out by the addition of 400 μl of supernatant to 1 × 10^6^ GXR cells in 1 ml R10 medium. The percent GFP (GFP%) was measured after 48 h to calculate the MOI.

The VRC assay was then carried out based on titration by infecting 1 × 10^6^ GXR cells in the same way as described above. The mean slope of exponential growth from days 2 to 7 was calculated using the semi-log method in Excel. This was divided by the slope of growth of the wild-type NL4-3 control included in each assay to generate a normalized measure of replication capacity. Replication assays were performed at least in duplicate, and results were averaged.

### Sequencing and phylogenetic analysis.

Plasma and viral stock sequencing was performed as described previously (33). Sequencing was undertaken using the BigDye Ready Reaction Terminator mix (V3.1) (Applied Biosystems, United Kingdom). Sequences were analyzed using Sequencher v4.8 (Gene Codes Corporation) and aligned by SeAl to the clade B reference strain HXB2. Estimation of founder sequences for the Mexico and Barbados cohorts was undertaken by the reconstruction of maximum likelihood phylogenetic trees using the FastML web server (34).

### Statistical analysis.

Comparisons between independent groups were performed using the Mann-Whitney U test for nonnormally distributed data or using the Student *t* test for normally distributed data; *P* values of <0.05 were considered significant. HLA and viral sequence variant frequencies were compared between groups using Fisher's exact test. The relationships between replication capacity and log viral load and CD4^+^ T-cell count were assessed using Spearman's rank correlation. Analyses were performed in GraphPad Prism (version 5.03) software (GraphPad Software, La Jolla, CA).

## RESULTS

### Lower viral loads and higher CD4^+^ T-cell counts observed in the Barbados cohort.

We first compared the viral loads and CD4^+^ T-cell counts from the two ART-naive clade B virus-infected cohorts (Barbados, *n* = 190 subjects; Mexico, *n* = 926). Although the same WHO guidelines for ART initiation have been followed in the two countries (27, 28), we observed a 2.9-fold difference in the viral loads (median, 14,200 versus 40,774 RNA copies/ml plasma in Barbados and Mexico, respectively; *P* < 0.0001) and a more modest difference in the CD4^+^ T-cell counts between cohorts (median, 403 and 380 cells/mm^3^ in Barbados and Mexico, respectively; *P* = 0.007) (see Fig. S1 in the supplemental material). This difference in viral loads may potentially have arisen from a variety of causes, including the possibility that ART was initiated later in the course of infection in Mexico than in Barbados. To further examine the potential contribution to this result of cohort-specific differences in viral replicative capacity, we therefore matched study subjects from each cohort for CD4^+^ T-cell count (see Materials and Methods) before determining the VRC in each.

### Relationship between viral replicative capacity, CD4^+^ T-cell counts, and viral load.

**FIG 2:**
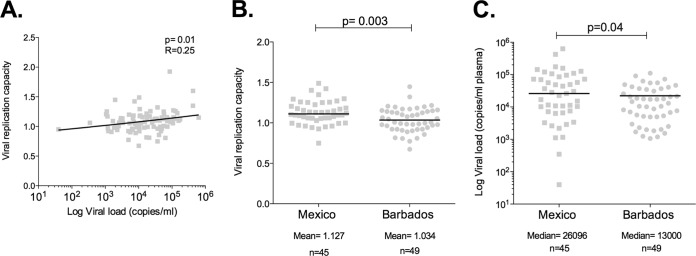
Viral replication capacities in Mexico and Barbados cohorts. (A) Correlation of replication capacity of chimeric Gag-Pro/NL4-3 with viral load in study subjects from both cohorts. (B) Viral replication capacities (VRC) in subjects from Mexico and Barbados. (C) Viral loads in subjects from Mexico and Barbados in whom VRC was determined.

In several previous studies that have employed the same method as the one adopted here to measure VRC, significant associations have consistently been observed between increasing viral load and increasing VRC and between increasing CD4^+^ T-cell count and decreasing VRC (8, 24, 32, 35–38). Consistent with these previous studies, in the pooled data from these two cohorts (*n* = 94), we observed a significant positive correlation between VRC and VL (*r* = 0.25; *P* = 0.02; Fig. 2A). There was no significant negative correlation between *in vitro* VRC and CD4^+^ T-cell counts in these subjects (*r* = −0.14; *P* = 0.19) selected across a relatively narrow range of CD4 count.

### Gag-protease chimeric viruses from Barbados have a lower RC than those from Mexico.

**FIG 3:**
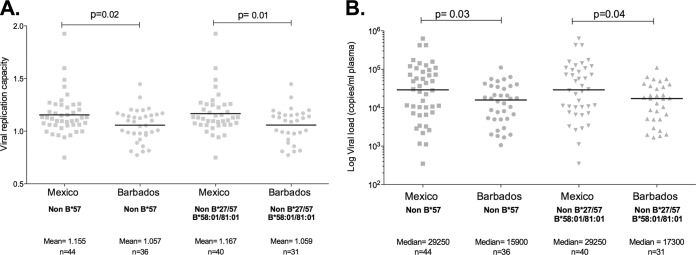
Viral replicative capacity and viral load in study cohorts excluding subjects expressing the protective HLA alleles HLA-B57/58:01/81:01/27. (A) Comparison of VRC in subjects from Barbados and Mexico lacking the protective HLA allele. (B) Comparison of viral loads in subjects lacking the protective HLA alleles in each cohort.

To avoid bias caused by disease progression, VRC was determined in each cohort in a subset of individuals whose CD4^+^ T-cell counts ranged between 300 and 500 cells/mm^3^ (mean CD4^+^ T-cell counts, 395 versus 389; *P* = 0.65). Consistent with our hypothesis, the mean VRC in Mexico was significantly higher than in Barbados (mean, 1.13 versus 1.03) (Fig. 3A). Furthermore, although the comparison groups were closely matched for CD4^+^ T-cell counts, viral loads remained significantly higher in the Mexican subset (median, 26,096 versus 13,000; *P* = 0.03) (Fig. 3B).

**FIG 4:**
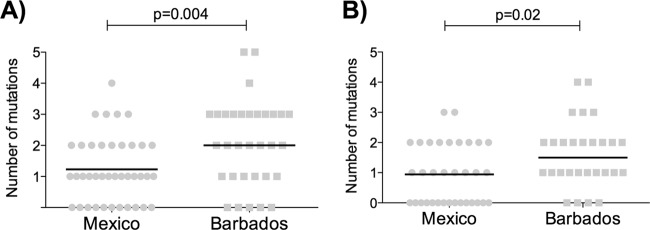
Frequency of Gag escape mutants associated with fitness cost in Mexico and Barbados study subjects. (A) Frequency of selected Gag escape variants in Mexican and Barbados populations (see the text). (B) Frequency of the same variants in the two cohorts but with the analysis limited to individuals not expressing the protective HLA molecules HLA-B*27/57/58:01/81:01.

To determine whether the difference in VRC was observed only due to the high frequency of individuals expressing protective alleles in Barbados, we repeated the analysis in the absence of individuals carrying protective alleles. VRC remained significantly higher in the Mexican cohort (*P* = 0.01) (Fig. 4A). Additionally, the median VL remained significantly higher in the Mexico cohort in the HLA-B*27/57/58:01/81:01-negative individuals (*P* = 0.04) (Fig. 4B).

### Accumulation of Gag escape mutations with viral fitness cost in Barbados.

To determine whether the differences in VRC observed between our study cohorts were associated with the accumulation of any of 8 Gag escape mutations previously shown to carry a fitness cost, namely, A146X, I147X, A163X, T186X, T242X, G248X, R264X, and T310X (4, 5, 7–18, 39), we analyzed the Gag sequences of the viruses included in the VRC measurements. As anticipated, the frequency of these HLA-B*27/57/58:01/81:01-associated escape mutations was significantly higher in the viruses generated from the Barbados cohort (*P* = 0.004 [Fig. 4A]), where the prevalence of these HLA alleles is substantially higher (Fig. 1).

To determine whether the differences that we observed between the two cohorts in median viral loads and viral replication capacity might be related in part to differences in the founder viruses in Barbados and Mexico, maximum likelihood phylogenetic trees were constructed for each cohort with the aim of estimating the founder virus in each case (see Table S1 in the supplemental material). They show that the estimated ancestral sequence for the Barbados and Mexico sequences differed by 7 amino acids within Gag, including I147L, which is one of the HLA-B*57-associated mutants. We therefore excluded the I147L mutant from the analysis to determine whether the differences in viral replicative capacity observed in Barbados and Mexico could be explained by this estimated founder effect. We observed that there was still a significantly greater number of escape mutants associated with the protective HLA alleles HLA-B*27/57/58:01/81:01 in the Barbados sequences analyzed for viral replicative capacity, even in subjects not expressing these particular protective HLA alleles (mean, 1.5 versus 1.0; *P* = 0.02) (Fig. 4B). Thus, the differential viral replicative capacities observed in Barbados and Mexico are not explained by HLA-B*27/57/58:01/81:01-associated mutation differences in founder viruses.

## DISCUSSION

The outcome of the transmission of escape mutations is not immediately intuitive. It might be expected that escape mutations that have a significant cost on VRC would rapidly revert when selection pressure is lost on transmission to an HLA-mismatched host (7, 40). However, in practice, reversion in the mismatched host takes time (41), and in some instances it does not occur at all (19) if either selection pressure for reversion is too weak (36) or compensatory mutants are sufficiently effective to reduce the fitness cost of the escape mutant (11, 29). In a population where the frequency of protective alleles is high, therefore, it is likely that the viruses transmitted most frequently from individuals expressing protective alleles will include those with escape mutations that reduce VRC, and this may result in those variants persisting at high levels in the population.

The present study examined the potential impact of a high frequency of protective HLA class I molecules on VRC at a population level. We hypothesized that this could lead to the accumulation of escape mutations with a fitness cost at a population level and therefore to a lower VRC in the population than in a cohort in which the frequency of protective alleles is lower. We compared population VRC in two HIV clade B-infected cohorts in Mexico and Barbados, in which the well-established protective HLA alleles, HLA-B*27/57/58:01/81:01, are expressed at very different frequencies, 8% and 34%, respectively. Consistent with this hypothesis, we observed that Mexican subjects, matched for CD4 count with subjects from the Barbados cohort, on average had virus exhibiting a higher viral replicative capacity (*P* = 0.0025) (Fig. 3). This difference remained significant even when patients with protective alleles were removed from the analysis and even after taking into account the potential role of founder viruses carrying escape mutants. This supports the hypothesis that a relatively low prevalence of protective HLA molecules, such as exists in Mexico, may contribute to a higher population level viral fitness, while a high prevalence of protective HLA alleles, as in Barbados, may contribute to a low population level viral fitness. These data are consistent with the observed high viral loads in ART-naive individuals in Mexico and relatively low viral loads in subjects in Barbados (28). Moreover, significantly higher viral loads were maintained in the Mexican subjects despite being matched by CD4^+^ T-cell count to the Barbados subjects and even in those subjects lacking the protective alleles B*27/57/58:01/81:01.

The differences in VRC observed between the two cohorts were associated with a higher frequency in the Barbados cohort of Gag variants previously shown to reduce VRC (B*57-A146X, B*57-A147X, B*57/58:01-T242N, B*57-G248A, B*27-R264K, and B*58:01-T310X), and this difference remained significantly higher in the Barbados cohort even in comparison of subjects not expressing these protective HLA alleles. For these analyses, we included HLA-B*27 as well as HLA-B*57/58:01/81:01, although the prevalence of the former is slightly higher in Mexico, since this is a well-established protective allele driving the selection of escape mutants that reduce viral replicative capacity (12, 13). The observed lower viral replicative capacity of viruses in Mexico versus Barbados therefore occurred in spite of a slightly higher frequency of HLA-B*27-associated escape mutants in the Mexico cohort (not shown), and reanalysis of the data having removed the B*27-associated data did not weaken the statistical significance of the findings (not shown). These data are consistent with the notions that escape mutations causing reduced VRC can be transmitted and remain stable in the population in spite of some fitness cost and that the higher prevalence of protective alleles in Barbados may at least in part contribute to the lower population level viral loads there than those observed in Mexico.

The observation that viral escape mutants are accumulating in Barbados suggests that the protective effect of alleles such as HLA-B*57 and HLA-B*58:01 may decline over time, as has been described for HLA-B*51 in Japan (19). HLA-B*57:03, the most prevalent of the HLA-B*57 alleles in Barbados, remains protective according to a recent study (30), although in Botswana we have observed loss of any protective effect mediated by these two closely related alleles (HLA-B*57/58:01) (R. Payne, P. Matthews, T. Ndung'u, S. Buus, B. D. Walker, and P. Goulder, unpublished data). Since the HIV seroprevalence in Botswana has been 20- to 30-fold higher than that in Barbados over the course of the epidemic, this factor is likely to have accelerated to a process of viral adaptation and abrogation of the protective effect of alleles such as HLA-B*57/58:01. The Mexico and Barbados epidemics arose at a similar time in the 1980s (42) and have operated at a somewhat lower adult seroprevalence in Mexico than in Barbados (0.2% in 2012 [www.unaids.org] compared to 0.8 to 1.1% in Barbados). Earlier studies in Amsterdam, Netherlands (43), suggested that viral adaptation was resulting in rapid epitope loss within HLA-B*27/57-restricted CTL epitopes over a 20-year period, in a locality where overall adult seroprevalence has not been dissimilar to that in Barbados and Mexico. In contrast, recent analyses of viral adaptation in North America suggest a low rate of viral adaptation (44). Thus, factors in addition to adult seroprevalence are likely to play a role in the rate of viral adaptation at the population level.

Potential shortcomings of the study are, first, that the platforms for measuring the viral loads in each cohort were not identical. In this study, the Abbott m2000 assay was used to measure viral load in the Mexican cohort starting in 2009, whereas in Barbados and before 2009 in Mexico, viral load measurements were performed with the Roche Cobas Amplicor and Roche AmpliPrep and TaqMan 48 Analysers. Viral load comparisons have shown overall strong agreement between these platforms; however, viral loads were reported consistently lower by the Abbott m2000 assay (45, 46). So, in our data set the viral load results in the Mexican cohort may in fact have been underestimated using the Abbott m2000 assay, and the differences between the two cohorts may in reality be greater than those observed. Second, it is possible that ART was initiated earlier in the course of disease in the Barbados cohort, which would provide an alternative explanation for the observed higher viral loads in Mexico. However, in fact the policies for ART initiation in both countries have followed WHO recommendations, and the CD4^+^ T-cell counts of the two cohorts were not greatly dissimilar (median, 380/mm^3^ versus 403/mm^3^). Furthermore, even once CD4 counts were matched, VRC was still significantly higher in the Mexican subjects.

A second shortcoming of the study is that we are measuring the replicative capacity of chimeric viruses that incorporate the Gag-Pro sequence from each study subject into NL4-3 and therefore any differences in replication resulting from changes to viral sequence elsewhere in the genome are not taken into account by this assay.

The data presented in the study are the first suggesting that a high frequency of protective alleles can shape viral evolution sufficiently to generate an alteration in VRC at a population level, in association with the accumulation of a higher number of HLA-associated Gag escape mutants. The viral set points and disease progression rates may therefore differ between distinct populations as a result of the frequency of protective alleles in the respective populations. In this case, the goal of achieving population level control of the HIV epidemic through initiation of ART at specific CD4 counts (currently at CD4 counts of ≤500/mm^3^) as recommended by the WHO is likely to be more problematic in some populations than others. On the basis that HIV transmission risk is related to viral load (47), consideration might therefore usefully be given to initiating ART at higher CD4^+^ T-cell counts in some populations than in others to optimize the impact of ART treatment for prevention.

## Supplementary Material

Supplemental material
