# Supplementary material for: Impact of HLA Selection Pressure on HIV Fitness at a Population Level in Mexico and Barbados
Source: J Virol. 2014 Sep;88(18):10392–8. doi: 10.1128/JVI.01162-14 (PMC4178877; doi:10.1128/JVI.01162-14)
Supplement: Supplemental material [file supp_88_18_10392__index.html]

Impact of HLA Selection Pressure on HIV Fitness at a Population Level in Mexico and Barbados — Supplemental material 

# Impact of HLA Selection Pressure on HIV Fitness at a Population Level in Mexico and Barbados

## Supplemental material

**Files in this Data Supplement:**

- Supplemental file 1 -

  Fig. S1 (Comparison of viral loads and CD4+ T-cell counts in the Mexico and Barbados cohorts.)

  Table S1 (Differences from the HXB2 Gag amino acid sequence of estimated ancestral viruses in Mexico and Barbados.)

  PDF, 109K
