# Supplementary material for: Impact of HLA Selection Pressure on HIV Fitness at a Population Level in Mexico and Barbados
Source: J Virol. 2014 Sep;88(18):10392–8. doi: 10.1128/JVI.01162-14 (PMC4178877; doi:10.1128/JVI.01162-14)
Supplement: Supplemental material [file JVI.01162-14_zjv999099512so1.pdf]

Supplementary Figure S1

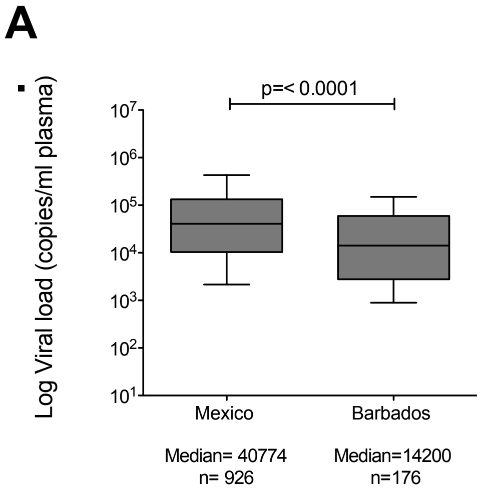

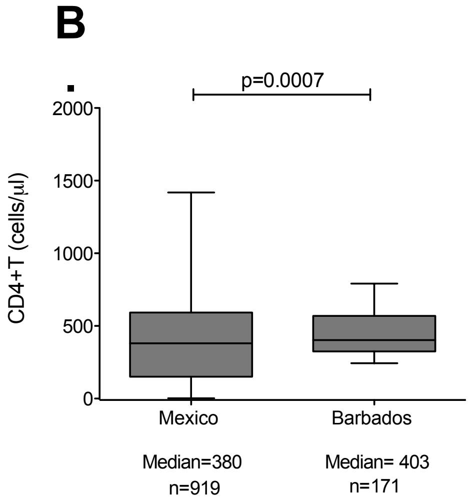

Supplementary Figure 1. Comparison of viral loads and CD4+T-cell counts in the Mexico and Barbados cohorts. A Viral loads. B. CD4+T-counts.

Supplementary table S1

Differences from HXB2 of estimated ancestral Gag amino acid sequence in Barbados and in Mexico

HXB2 Gag	30	76	84	94	124	126	138	147	159	215	280	312
HXB2 Sequence	K	R	T	I	H	N	I	I	V	V	T	E
Barbados	-	K	V	V	N	S	L	L	I	L	V	D
Mexico	R	V	V	V	N	-	L	-	-	L	-	-

Supplementary Table 1. **Differences from HXB2 Gag amino acid sequence of estimated ancestral viruses in Mexico and Barbados**.
